# Atypical Presentation of Fournier Gangrene as Syncope in a Patient With Chronic Alcohol Use Disorder: A Case Report

**DOI:** 10.7759/cureus.113187

**Published:** 2026-07-22

**Authors:** Anvitha Kambham, Brendan Masi, Abraham E Libman, Himanshukumar Nayak, Roxana Lazarescu

**Affiliations:** 1 Internal Medicine, Touro College of Osteopathic Medicine, New York, USA; 2 Internal Medicine, Wyckoff Heights Medical Center, New York, USA

**Keywords:** alcohol use disorder, computed tomography, fournier gangrene, immune dysfunction, necrotizing fasciitis, perianal abscess, sepsis, soft tissue infection, surgical debridement, syncope

## Abstract

Fournier gangrene is a rapidly progressive necrotizing soft tissue infection of the perineal and genital regions associated with substantial morbidity and mortality. Chronic alcohol use disorder (AUD) is a recognized predisposing condition because of its effects on immune function, nutritional status, and hepatic function. We report a 63-year-old man with AUD who presented after a syncopal episode with abdominal pain and systemic inflammatory response syndrome criteria but without a documented perineal or genital complaint. Computed tomography (CT) of the abdomen and pelvis demonstrated soft tissue gas surrounding the anorectal junction and extending into the perineum, concerning for Fournier gangrene. The patient underwent urgent incision and drainage of a perianal abscess, and wound cultures grew *Klebsiella oxytoca* and *Raoultella ornithinolytica*. Evaluation did not identify diabetes mellitus, human immunodeficiency virus infection, or the other evaluated predisposing conditions. No primary neurologic or cardiac cause of syncope was identified, and the systemic effects of sepsis were considered the most likely explanation. This case illustrates that Fournier gangrene may present with systemic symptoms in the absence of localizing perineal complaints. Prompt examination, imaging, surgical evaluation, and antimicrobial therapy remain essential.

## Introduction

Fournier gangrene is an uncommon but aggressive type of necrotizing fasciitis involving the perineal, perianal, genital, and scrotal regions. The infection rapidly spreads through the superficial and deep fascial planes, causing extensive inflammation, tissue necrosis, sepsis, and substantial morbidity. Despite treatment, it carries a mortality rate approaching 40% [1]. Many conditions that impair host immunity are associated with Fournier gangrene, including diabetes mellitus, chronic alcohol use disorder (AUD), human immunodeficiency virus (HIV) infection, immunosuppression, and malignancy [2]. Fournier gangrene is typically a polymicrobial infection involving a combination of aerobic and anaerobic bacteria. Frequently isolated aerobic pathogens include *Escherichia coli*, *Klebsiella*, *Proteus*, *Staphylococcus*, and *Streptococcus *species. Common anaerobic organisms include *Bacteroides*, *Clostridium*, and *Peptostreptococcus *species [3]. The pathologic process may begin with infection or local tissue injury, including urinary tract infection, perineal abscess, cellulitis, recent instrumentation or surgery involving the genital or perineal region, or minor skin trauma. The interaction between aerobic and anaerobic organisms leads to the release of destructive enzymes, collagenases, and endotoxins that damage surrounding tissue and blood vessels. This process causes obliterative endarteritis and microvascular thrombosis, resulting in tissue ischemia, gangrene, and rapid extension of infection [1].

Because Fournier gangrene can quickly progress to fulminant infection, prompt diagnosis and source control are essential. Computed tomography (CT) is the most specific imaging technique for determining the extent of infection and can assist the surgical team in planning debridement. Radiography and ultrasonography may also contribute to diagnosis [4]. Treatment includes broad-spectrum antimicrobial therapy and urgent surgical debridement, with negative-pressure wound therapy, vacuum-assisted closure, or hyperbaric oxygen therapy used in selected cases [4]. Classic manifestations include perineal pain, swelling, erythema, crepitus, skin discoloration, and systemic toxicity, although localizing symptoms or findings may be subtle early in the disease course. Syncope as the presenting complaint without a documented perineal or genital complaint is unusual and may initially direct attention toward neurologic or cardiac causes. Chronic alcohol use may further increase susceptibility through impaired immune function, nutritional compromise, hepatic dysfunction, and impaired wound healing [5,6]. We report a patient with AUD who presented after syncope and was subsequently found on CT to have Fournier gangrene. The educational value of this case lies in considering an occult perineal source in septic patients with relevant risk factors even when localizing complaints are absent.

## Case presentation

A 63-year-old man with a history of AUD presented to the emergency department (ED) after losing consciousness while standing in a store. He struck his head, reportedly lost consciousness for approximately two minutes, and had bowel incontinence. He reported left-sided abdominal and right upper quadrant pain but denied chest pain, fever, chills, palpitations, shortness of breath, nausea, vomiting, urinary incontinence, tongue biting, or other seizure-like activity. No perineal or genital complaint was documented at presentation. He appeared jaundiced and reported drinking approximately one to two eight-ounce cups of whisky almost every day for 30 years.

In the ED, he met systemic inflammatory response syndrome (SIRS) criteria based on a heart rate greater than 90 beats per minute (bpm) and a white blood cell (WBC) count greater than 12,000/mm^3^. He was afebrile, had a heart rate of 132 bpm, and had a WBC count of 12.7 × 10^3^/µL. A detailed initial examination of the perineal and genital regions and the findings of any digital rectal examination could not be confirmed from the records available to the authors. Other admission laboratory findings are presented in Table 1. Urinalysis and urine culture were unremarkable. Empiric clindamycin, piperacillin-tazobactam, and vancomycin were initiated. He also received intravenous hydration, thiamine, folic acid, and electrolyte supplementation as needed.

**Table 1:** Patient’s lab values on initial presentation to the hospital and on discharge. WBC, white blood cell count; RBC, red blood cell count; MCV, mean corpuscular volume; MCH, mean corpuscular hemoglobin; MCHC, mean corpuscular hemoglobin concentration; RDW, red cell distribution width; CO_2_, serum bicarbonate (total carbon dioxide); BUN, blood urea nitrogen; AST, aspartate aminotransferase; ALT, alanine aminotransferase; eGFR, estimated glomerular filtration rate. The Laboratory Risk Indicator for Necrotizing Fasciitis (LRINEC) score [7] could not be calculated retrospectively because a C-reactive protein (CRP) value was unavailable. The Fournier Gangrene Severity Index (FGSI) [8] could not be calculated reliably because the exact admission temperature and respiratory rate were not available in the records reviewed. A serum lactate value was also unavailable.

Lab	Patient values on admission	Patient values on discharge	Normal range
WBC	12.7 ↑	8.5	4.5–11.0 x 10^3^/µL
RBC count	4.26 ↓	—	Male: 4.3–5.9 x 10^6^/µL
Hemoglobin	13.2	—	Male: 13.5–17.5 g/dL
Hematocrit	39.1	—	Male: 41–53%
MCV	91.8	—	80–100 fL
MCH	31.0	—	26–34 pg
MCHC	33.8	—	32–36 g/dL
RDW	12.7	—	11.5–14.5%
Platelet count	52 ↓	384	150–400 x 10^3^/µL
Neutrophils	84.6 ↑	59.9	54–62%
Lymphocytes	6.8 ↓	—	25–33%
Monocytes	5.7	—	3–7%
Eosinophils	0.0 ↓	—	1–3%
Basophils	0.3	—	0–0.75%
Immature granulocytes	2.6 ↑	—	0–0.5%
Acetaminophen level	<2.0	—	Therapeutic: 10–30 µg/mL
Calcium	7.9 ↓	8.5	8.4–10.2 mg/dL
Albumin	2.5 ↓	2.7 ↓	3.5–5.0 g/dL
Total protein	5.8 ↓	6.8	6.0–8.0 g/dL
Sodium	131 ↓	139	136–145 mEq/L
Potassium	3.3 ↓	3.9	3.5–5.0 mEq/L
Chloride	92 ↓	—	95–105 mEq/L
CO_2_	31	—	23–30 mEq/L
BUN	17	—	7–18 mg/dL
Glucose	132	109	<140 mg/dL, non-fasting
Total bilirubin	12.9 ↑	3.7 ↑	0.1–1.0 mg/dL
Direct bilirubin	10.58 ↑	3.27 ↑	0.0–0.3 mg/dL
Alkaline phosphatase	242 ↑	597 ↑	45–115 U/L
AST	467 ↑	276 ↑	10–40 U/L
ALT	294 ↑	292 ↑	7–56 U/L
Creatinine	1.59 ↑	—	Male: 0.6–1.2 mg/dL
eGFR	48 ↓	—	>60 mL/min/1.73 m^2^

**Figure 1:**
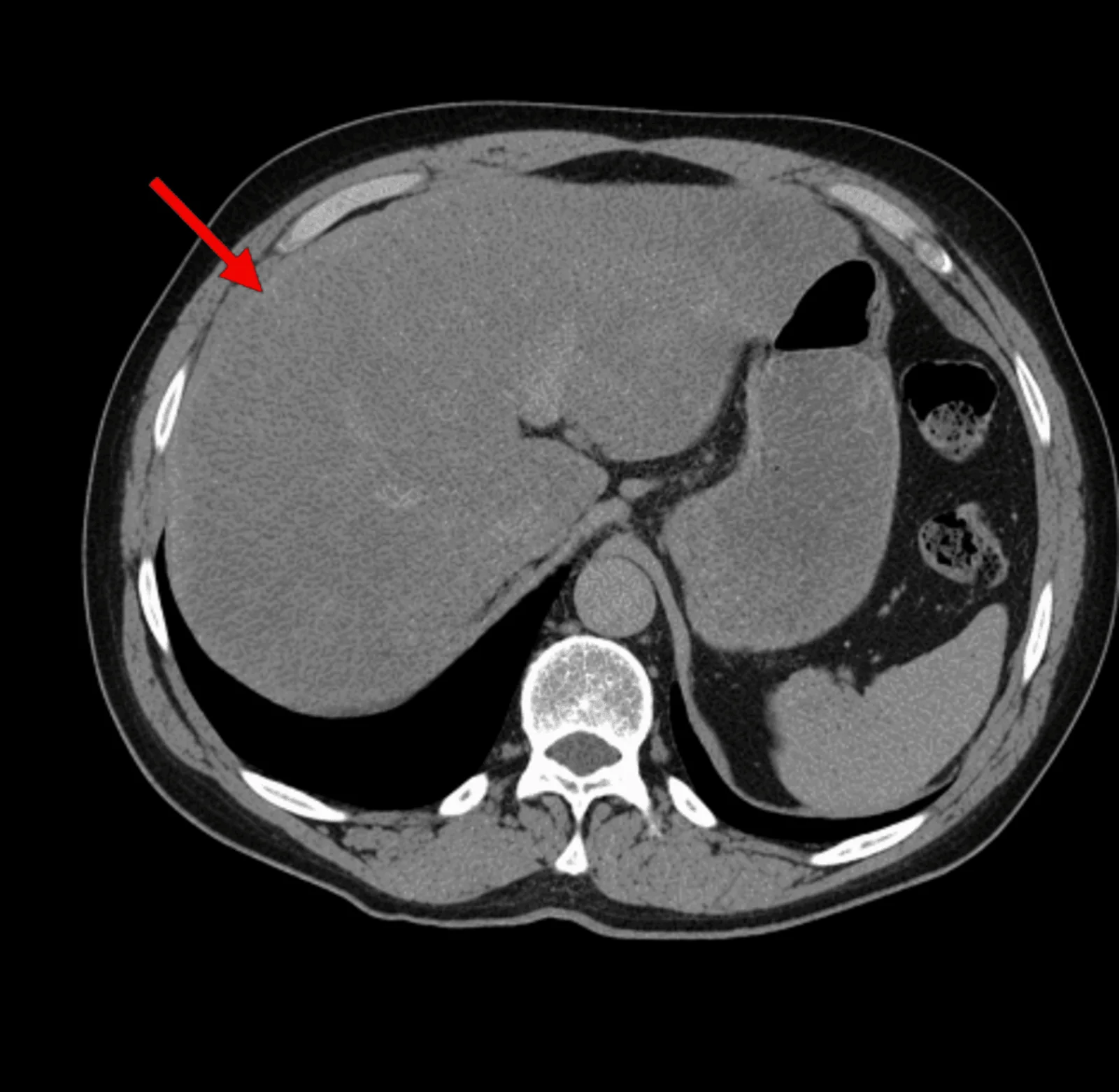
Computed tomography of abdomen and pelvis without contrast. Computed tomography (CT) of the abdomen and pelvis without contrast showing hepatomegaly with diffuse low hepatic attenuation consistent with hepatic steatosis; the liver measured 19.9 cm (red arrow).

Emergent imaging was obtained. Head CT showed no acute intracranial abnormality, and chest radiography showed no acute pulmonary process. CT of the abdomen and pelvis showed hepatomegaly with diffuse low hepatic attenuation consistent with hepatic steatosis; the liver measured 19.9 cm (Figure 1).

**Figure 2:**
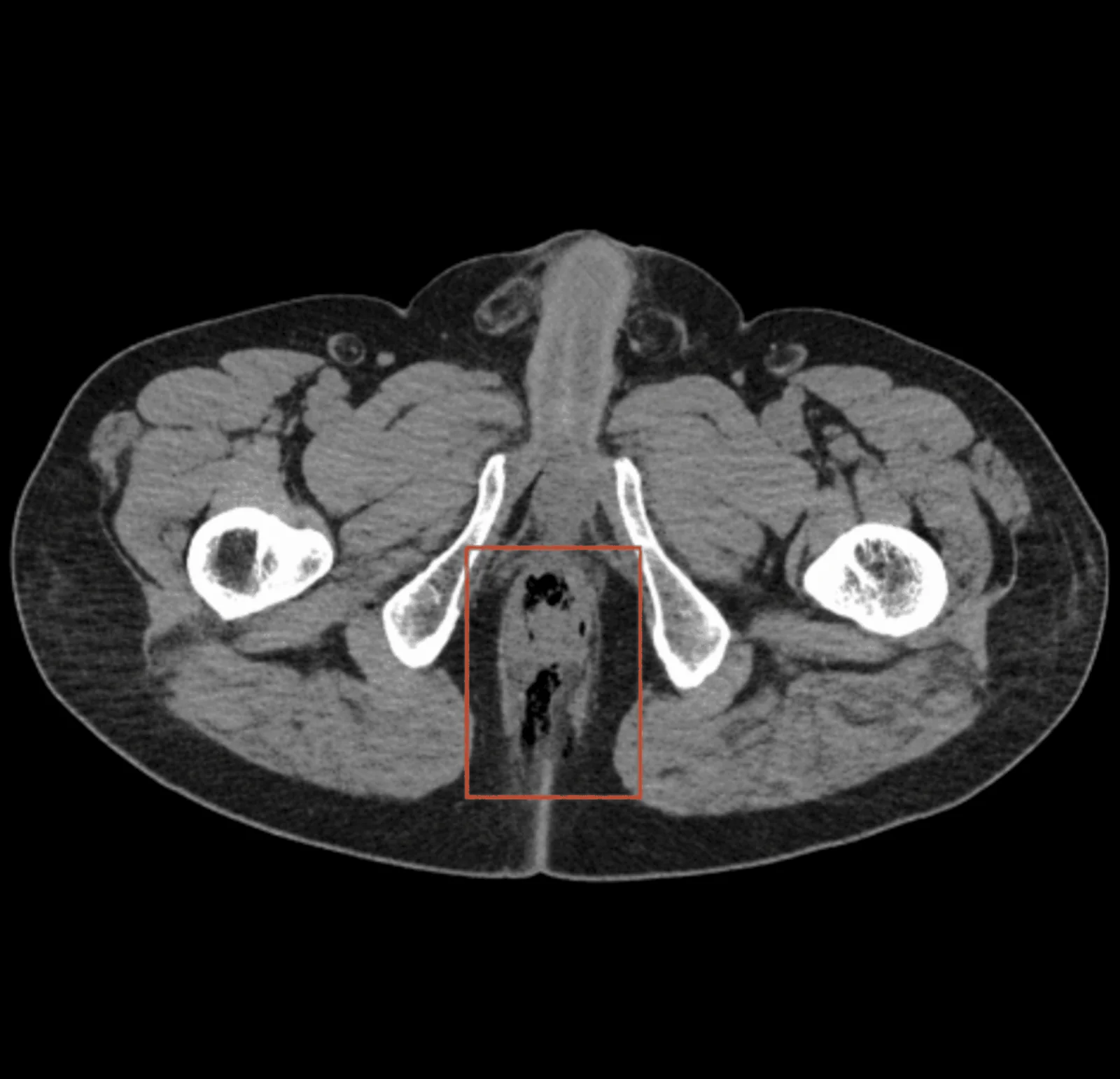
Axial computed tomography of the abdomen and pelvis. Axial computed tomography (CT) image of the abdomen and pelvis showing soft tissue gas surrounding the anorectal junction and extending into the perineum (red box), concerning for Fournier gangrene.

**Figure 3:**
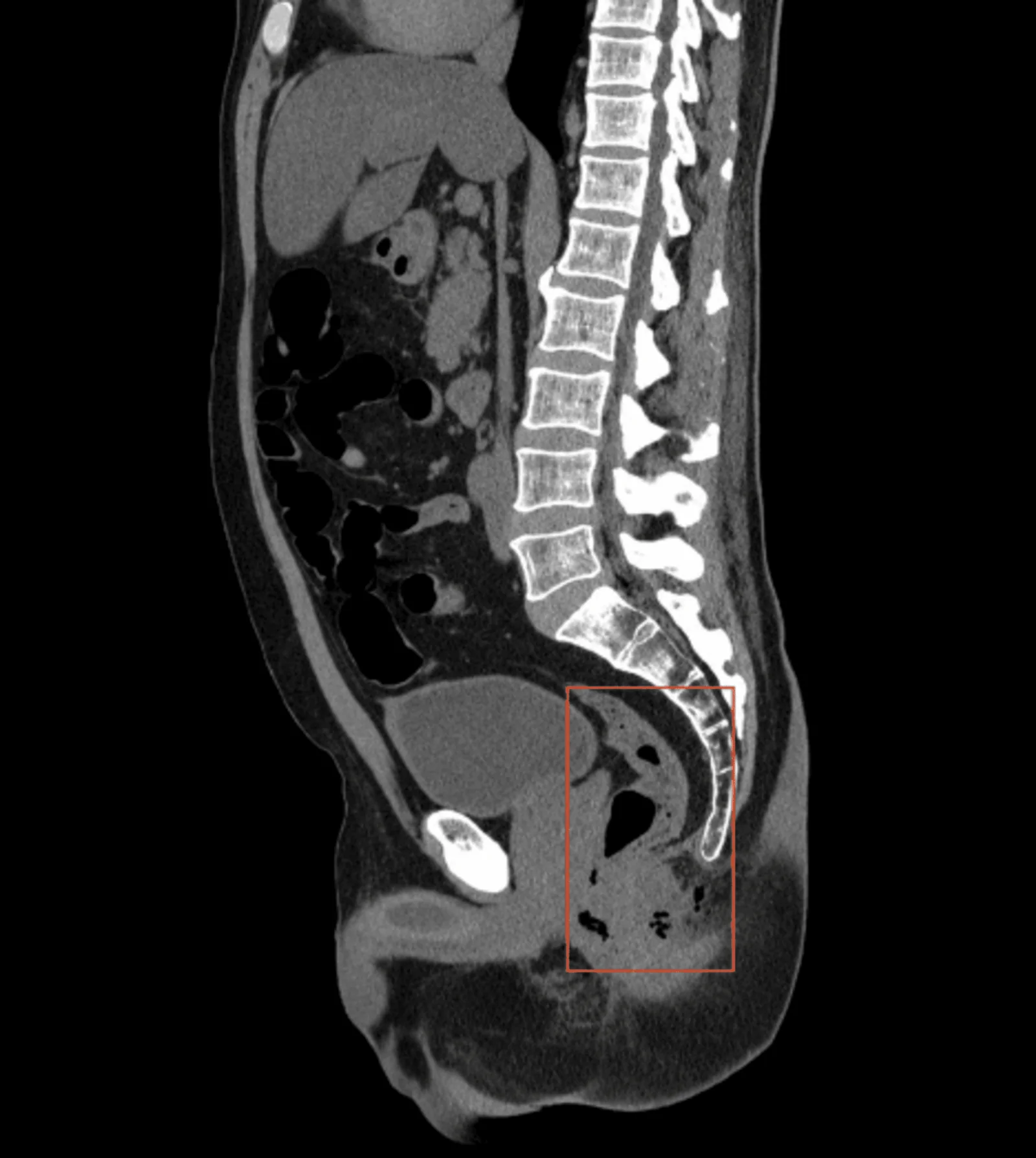
Sagittal computed tomography of the abdomen and pelvis. Sagittal computed tomography (CT) image of the abdomen and pelvis showing soft tissue gas extending into the perineum (red box), concerning for Fournier gangrene.

Axial and sagittal images also demonstrated soft tissue gas surrounding the anorectal junction and extending into the perineum, concerning for Fournier gangrene (Figures 2, 3). The exact intervals from ED arrival to CT and from CT to the operating room (OR) could not be confirmed from the records available to the authors.

The patient underwent incision and drainage of a perianal abscess, and serosanguineous purulent fluid was sent for culture. The operative information available to the authors did not include detailed findings from examination under anesthesia, the extent or depth of necrotic tissue, the extent of tissue excision or debridement, or whether reconstruction was required. Cultures grew *Klebsiella oxytoca* (*K. oxytoca*) and *Raoultella ornithinolytica *(*R. ornithinolytica*), and Gram staining showed rare Gram-positive cocci in pairs. The infectious diseases and pharmacy teams recommended discontinuing the initial antimicrobial regimen and beginning ceftriaxone and metronidazole.

Neurology and cardiology were consulted to evaluate the syncopal episode. A 12-hour electroencephalogram (EEG) showed no seizure activity, carotid ultrasonography was unremarkable, and electrocardiography (ECG) showed sinus tachycardia. Echocardiography demonstrated a left ventricular ejection fraction (LVEF) of 55%-60% with grade 1 diastolic dysfunction. No primary neurologic or cardiac cause of syncope was identified. Sepsis-associated transient cerebral hypoperfusion related to vasodilation, relative intravascular depletion, or autonomic instability was considered the most likely explanation. However, a serum ethanol concentration, orthostatic vital signs, and the exact time of the patient’s last alcohol use could not be confirmed from the available records; therefore, alcohol intoxication could not be excluded with certainty.

The patient denied a history of anal intercourse or anal penetration. Testing for sexually transmitted infections (STIs), including HIV, syphilis, chlamydia, and gonorrhea, was negative. Hemoglobin A1c was 6.0%. Antinuclear antibody (ANA) testing with reflex to titer and pattern and mitochondrial antibody testing were negative. This evaluation did not identify diabetes mellitus, HIV infection, the tested STIs, or the tested autoimmune conditions as alternative predisposing factors.

At discharge, the WBC count and platelet count had normalized. Liver enzymes, total and direct bilirubin, and alkaline phosphatase remained elevated, as shown in Table 1. The patient was afebrile with a heart rate of 84 bpm, and the remaining documented vital signs were stable. The records available to the authors did not include a detailed wound assessment at discharge, the outpatient wound-care plan, whether repeat debridement or reconstruction was required, or subsequent surgical follow-up. The patient was discharged with planned hepatology follow-up and resources for alcohol cessation. Longer-term clinical and wound outcomes were unavailable.

## Discussion

Chronic alcohol use can disrupt host defense through several complementary mechanisms. Alcohol metabolism generates reactive oxygen species (ROS), which can activate nuclear factor kappa B (NF-κB) and promote inflammatory signaling involved in alcohol-associated liver injury [5]. Chronic alcohol exposure can also disturb the balance between proinflammatory and anti-inflammatory responses within the innate immune system. It impairs neutrophil chemotaxis, phagocytosis, and oxidative burst activity, reducing the ability to contain bacterial infection [5]. Adaptive immunity may also be affected through reduced lymphocyte number and altered dendritic-cell activation of T cells [5]. In addition, chronic alcohol use can alter the intestinal microbiome, favor gram-negative bacterial overgrowth, and impair wound healing through disruption of inflammatory-cell function, collagen production, angiogenesis, and re-epithelialization [5,6].

These mechanisms are clinically relevant to this case because the patient developed a severe enteric soft tissue infection in the setting of prolonged alcohol use, hepatic dysfunction, and possible nutritional compromise. He had thrombocytopenia, hypoalbuminemia, hyperbilirubinemia, elevated transaminases, hepatomegaly, and hepatic steatosis, supporting chronic alcohol-associated hepatic injury. Testing did not identify diabetes mellitus, human immunodeficiency virus infection, the evaluated sexually transmitted infections, or the tested autoimmune conditions as alternative predisposing factors. Although these findings do not establish that alcohol use caused the infection, they support chronic alcohol use disorder as a plausible contributor to impaired host defense and disease severity in this patient.

Syncope was an unusual presenting complaint but was not truly isolated, because the patient also had abdominal pain, tachycardia, leukocytosis, and other evidence of systemic illness. No perineal or genital complaint was documented. Sepsis-associated syncope may result from transient cerebral hypoperfusion related to vasodilation, relative intravascular depletion, or autonomic dysfunction. In this patient, neurologic and cardiac evaluation did not identify an alternative cause, although alcohol intoxication could not be excluded with certainty because a serum ethanol concentration and exact time of last alcohol use were unavailable. Reviews describe early Fournier gangrene presenting with nonspecific pain, systemic toxicity, or minimal local findings before overt tissue changes become apparent [1,9]. CT was therefore critical in identifying perineal soft tissue gas and prompting surgical intervention. Because the detailed initial perineal examination and exact ED-to-CT and CT-to-OR intervals were unavailable, this report cannot determine whether local findings were absent or undocumented or whether a measurable diagnostic delay occurred.

Wound cultures grew *K. oxytoca* and *R. ornithinolytica*. *Klebsiella *species are recognized aerobic pathogens in Fournier gangrene [3], whereas *R. ornithinolytica* is not among the organisms commonly emphasized in the major reviews cited here and was therefore an unusual isolate in this report. Both organisms are compatible with an enteric source arising from the perianal infection. Surgical drainage, initial broad-spectrum antimicrobial therapy, and subsequent culture-directed treatment were followed by normalization of the WBC and platelet counts and stabilization before discharge.

In our patient, we hypothesize that chronic alcohol use was a probable predisposing factor for the development of Fournier gangrene. Although diabetes mellitus is the most commonly reported risk factor, chronic alcohol use disorder has also been identified as an independent contributor to Fournier gangrene through its effects on immune function, nutritional status, and hepatic dysfunction. Reviews of Fournier gangrene consistently identify alcohol misuse, malnutrition, diabetes mellitus, and immunosuppression as important predisposing conditions, supporting chronic alcohol use disorder as a clinically meaningful risk factor in this patient [10]. In a retrospective analysis of 25 patients with Fournier gangrene, alcohol misuse was recognized as one of the major predisposing conditions associated with disease development [11]. Several case reports have also described Fournier gangrene occurring in patients with severe alcohol-associated liver disease. For example, Zenda et al. reported Fournier gangrene in a patient with severe alcoholic hepatitis and concluded that the infection likely developed in the setting of impaired immunologic defenses caused by chronic alcohol consumption and advanced liver dysfunction [12]. In the present case, evaluation did not identify diabetes mellitus, human immunodeficiency virus infection, the evaluated sexually transmitted infections, or the tested autoimmune conditions as alternative predisposing factors. Together with the patient's prolonged alcohol use history and evidence of alcohol-associated liver injury, these findings support chronic alcohol use disorder as a plausible major contributor to the development of Fournier gangrene, although causation cannot be established from a single case.

This report is limited by the retrospective availability of clinical records. Detailed initial perineal, genital, and digital rectal examination findings; exact ED-to-CT and CT-to-OR intervals; serum lactate and C-reactive protein values; complete inputs for retrospective severity scores; detailed examination-under-anesthesia and debridement findings; postoperative wound management; reconstruction; serum ethanol and orthostatic data; and long-term surgical follow-up were unavailable. Consequently, the precise mechanism of syncope and the relative contribution of chronic alcohol use remain probable rather than proven.

## Conclusions

Fournier gangrene may first be identified during the evaluation of syncope and sepsis even when no perineal or genital complaint is documented. In this case, syncope was not truly isolated because abdominal pain and systemic inflammatory findings were also present. Chronic AUD with associated hepatic and possible nutritional dysfunction was a plausible predisposing factor, although causation could not be established. In septic patients with unexplained abdominal or perirectal symptoms and relevant risk factors, clinicians should perform a targeted perineal examination and maintain a low threshold for CT and surgical consultation. Prompt antimicrobial therapy and surgical source control remain essential.
